# Alloimmune Causes of Recurrent Pregnancy Loss: Cellular Mechanisms and Overview of Therapeutic Approaches

**DOI:** 10.3390/medicina60111896

**Published:** 2024-11-19

**Authors:** Cristina Uța, Alexandru Tîrziu, Elena-Larisa Zimbru, Răzvan-Ionuț Zimbru, Marius Georgescu, Laura Haidar, Carmen Panaitescu

**Affiliations:** 1 Center of Immuno-Physiology and Biotechnologies, Department of Functional Sciences, “Victor Babeș” University of Medicine and Pharmacy, 2 Eftimie Murgu Square, 300041 Timisoara, Romania; cristina.uta@umft.ro (C.U.); elena.zimbru@umft.ro (E.-L.Z.); razvan.zimbru@umft.ro (R.-I.Z.); georgescu.marius@umft.ro (M.G.); cbunu@umft.ro (C.P.); 2 Timis County Emergency Clinical Hospital “Pius Brinzeu”, 156 Liviu Rebreanu Bd., 300723 Timisoara, Romania; 3 Department of Functional Sciences, Physiology Discipline, Faculty of Medicine, “Victor Babeș” University of Medicine and Pharmacy Timișoara, 2 Eftimie Murgu Square, 300041 Timişoara, Romania; 4 Institute of Cardiovascular Diseases Timisoara, 13A Gheorghe Adam Street, 300310 Timisoara, Romania; 5 Research Center for Gene and Cellular Therapies in the Treatment of Cancer—OncoGen, Timis County Emergency Clinical Hospital “Pius Brinzeu”, 156 Liviu Rebreanu Bd., 300723 Timisoara, Romania

**Keywords:** recurrent pregnancy loss, alloimmune, NK cells, T cells, killer cell immunoglobulin-like receptors, immunotherapy

## Abstract

Recurrent pregnancy loss (RPL) is a complex early pregnancy complication affecting 1–2% of couples and is often linked to immune dysfunction. Aberrations in T and B cell subpopulations, as well as natural killer (NK) cell activity, are particularly influential, with studies showing that abnormal NK cell activation and imbalances in T and B cell subtypes contribute to immune-mediated miscarriage risk. Successful pregnancy requires a tightly regulated balance between pro-inflammatory and anti-inflammatory immune responses. In the early stages, inflammation supports processes such as trophoblast invasion and spiral artery remodeling, but this must be tempered to prevent immune rejection of the fetus. In this review, we explore the underlying immune mechanisms of RPL, focusing on how dysregulated T, B, and NK cell function disrupts maternal tolerance. Specifically, we discuss the essential role of uterine NK cells in the early stages of vascular remodeling in the decidua and regulate the depth of invasion by extravillous trophoblasts. Furthermore, we focus on the delicate Treg dynamics that enable the maintenance of optimal immune homeostasis, where the balance, and not only the quantity of Tregs, is crucial for fostering maternal–fetal tolerance. Other T cell subpopulations, such as Th1, Th2, and Th17 cells, also contribute to immune imbalance, with Th1 and Th17 cells promoting inflammation and potentially harming fetal tolerance, while Th2 cells support immune tolerance. Finally, we show how changes in B cell subpopulations and their functions have been associated with adverse pregnancy outcomes. We further discuss current therapeutic strategies aimed at correcting these immune imbalances, including intravenous immunoglobulin (IVIg), glucocorticoids, and TNF-α inhibitors, examining their efficacy, challenges, and potential side effects. By highlighting both the therapeutic benefits and limitations of these interventions, we aim to offer a balanced perspective on clinical applications for women facing immune-related causes of RPL.

## 1. Introduction

Recurrent pregnancy loss (RPL) is one of the most challenging and frustrating aspects of reproductive medicine as it can cause significant emotional distress and have a major impact on couples. Its underlying causes are often unclear, and diagnostic and therapeutic options are limited. According to the European Society of Human Reproduction and Embryology (ESHRE) and the American Society for Reproductive Medicine (ASRM), RPL affects 1–2% of couples and is defined as two or more, consecutive or not, pregnancy losses before 20–24 weeks of gestation [1,2]. The ESHRE states that the pregnancy must be confirmed through the detection of serum or urinary chorionic gonadotropin, and its definition specifically excludes cases of ectopic and molar pregnancies [3].

RPL presents significant physical and mental health challenges. For instance, it may contribute to fertility issues, as each event has the potential to damage the uterine lining, decreasing the likelihood of successful conception [4]. RPL also increases health risks in future pregnancies, including a higher likelihood of intrauterine adhesions, endometriosis, endometrial polyps, adenomyosis, uterine fibroids, and cervical insufficiency [5,6]. Additionally, the psychological impact on women can be profound, often leading to conditions such as anxiety, depression, and emotional distress, especially when the pregnancy was desired, although reactions can vary depending on individual circumstances, including the nature of the pregnancy [7]. Women experiencing RPL may grapple with feelings of grief, loss, and inadequacy, which can intensify with each successive miscarriage. The emotional toll is further compounded by the uncertainty surrounding the cause of their losses and the fear of future miscarriages. This psychological strain can interfere with daily functioning, impact relationships, and even discourage women from pursuing further pregnancies. Addressing these mental health concerns is a critical component of comprehensive care for women affected by RPL [8]. Moreover, men may also experience significant psychological effects following RPL, though their experiences are often less visible. Men may undergo intense grief with each loss, grappling with a diminished sense of their role as a father, and feel disheartened by their limited ability to actively influence or prevent the outcome. Studies suggest that men frequently feel a dual burden: managing their personal grief while striving to be a source of strength and support for their partners [9]. This role can be emotionally challenging and isolating, as men may feel societal or cultural pressure to suppress their own sorrow and prioritize their partner’s needs, leading to feelings of frustration and helplessness [10]. Men may also feel overlooked or unacknowledged by both family and medical professionals, as the focus tends to center on women’s experiences, often leaving men without adequate emotional support [8]. This lack of recognition can lead to a sense of disenfranchised grief, where men feel that their sorrow is somehow invalid or secondary [11,12]. As the frequency of losses increases, so does the emotional toll, intensifying feelings of inadequacy, despair, and, in some cases, even anger [13]. Beyond these individual impacts, the strain of RPL can extend to marital and familial relationships. Partners may experience increased tension and misunderstandings as each navigates grief in unique ways, sometimes resulting in communication breakdowns. These experiences of loss and the ongoing stress of fertility challenges can place a heavy strain on intimacy, trust, and mutual support within the relationship. Consequently, addressing the psychological impact of RPL from a holistic partner-inclusive perspective is essential to fully understand the broader emotional burden that recurrent losses bring to both individuals and their relationship as a whole [14,15]. Finally, the condition can result in significant financial burden, particularly if multiple investigative procedures and medical interventions become necessary [16].

There are several presumed etiologies of embryo rejection, including chromosomal abnormalities, genetic disorders, endocrine disorders, uterine malformations, thrombus-prone factors, infection, stress, and dysregulated maternal immunological tolerance [17]. However, in approximately 50% of RPLs, the underlying causes remain unidentified. Due to the idiopathic nature of these cases, research has increasingly focused on immunological risk factors [18].

To the maternal immune system, the embryo is perceived as a semi-allogeneic graft, as half of its genetic material comes from the father. A semi-allogeneic graft refers to tissue or cells that are genetically similar but not identical to those of the recipient. In the context of pregnancy, the embryo is considered semi-allogeneic because it contains both maternal and paternal genetic material. Half of the embryo’s genes are shared with the mother (making it partly “self”), while the other half, from the father, are “non-self” from the perspective of the mother’s immune system [19,20]. However, the maternal immune system must tolerate its presence to sustain a healthy pregnancy, a process that involves a delicate balance of immune regulation, where certain immune responses are suppressed to avoid attacking the fetus, while still maintaining overall immune function. Therefore, a highly specific immunological interaction between the embryo and the maternal immune system is necessary from the blastocyst stage to implantation and onwards [21]. Abnormalities of the mother’s immune system may cause the embryo to be considered a foreign entity and initiate an immune response, leading to pregnancy loss. Recent advancements in the understanding of immune mechanisms at the maternal–fetal interface have highlighted the complex interplay between innate and adaptive immune cells, including natural killer (NK) cells, T-helper (Th) cell subpopulations, and regulatory T cells (Tregs). Their dysregulation, particularly an overactivation of Th1 and Th17 cells or elevated NK cell cytotoxicity, can disrupt maternal–fetal tolerance and induce a cytotoxic environment in the uterus, inadequate angiogenesis, increased oxidative stress, or ischemic changes at the trophoblast level, all of which are part of the etiology of pregnancy loss [22]. Additionally, cytokine dysregulation and HLA incompatibilities between the mother and fetus have been identified as critical factors influencing immune tolerance.

Advances in diagnostic tools now allow for more precise assessments of immune factors, such as NK cell activity and cytokine profiles, which can inform personalized therapeutic approaches. Meanwhile, the use of immunomodulatory therapies, including intravenous immunoglobulin (IVIG), intralipid, and TNF-α inhibitors, has gained traction in clinical settings, with emerging data suggesting their potential in improving pregnancy outcomes for immunologically mediated RPL [23]. Furthermore, global trends in research emphasize the importance of population diversity, revealing how genetic and environmental factors influence immune responses and pregnancy outcomes [24]. The growing body of evidence on these mechanisms has paved the way for novel therapeutic strategies, such as immunomodulatory agents, to restore immune balance and improve pregnancy outcomes. Incorporating these insights is essential to developing more targeted and effective approaches to managing RPL [25,26].

The aim of the different treatment options for alloimmune causes of RPL is to improve pregnancy outcomes by decreasing RPL rates and improving the chances of live at-term births; however, the results so far have been modestly promising. Understanding the complex processes that lead to dysfunctions at a molecular and cellular level and contribute to RPL is crucial for developing more targeted and effective treatments. With this knowledge, therapies that specifically address these immune challenges can lead to significant improvements in pregnancy outcomes for women affected by these conditions, helping to ensure healthier pregnancies and reducing the emotional and physical toll of recurrent pregnancy loss.

## 2. Immune Cells at the Maternal–Fetal Interface

When sperm and seminal fluid enter the female reproductive system during intercourse, maternal immune cells in the reproductive tissues encounter paternal antigens for the first time. After fertilization in the fallopian tubes, the developing embryo, now called a blastocyst, moves down toward the uterus. As it continues to divide and grow, the blastocyst reaches the uterus and attaches to the decidua, the specialized uterine lining. During implantation, the blastocyst begins to embed itself in the uterine tissue, initiating early pregnancy [27].

At the maternal–fetal interface, immune cells such as NK, T, and B cells play critical roles in immune tolerance. The maternal immune system recognizes the paternal antigens from the seminal fluid, and different mechanisms, such as regulatory T and B cells and anti-inflammatory interleukins, need to be engaged in order to provide a tolerogenic environment. In the context of pregnancy, a tolerogenic environment refers to an immune state within the maternal body that promotes tolerance, allowing the maternal immune system to accept the semi-allogeneic fetus (which carries both maternal and paternal antigens) without mounting an immune response that would otherwise reject it. This tolerogenic state is established through complex interactions between the trophoblast cells, such as extravillous trophoblasts (EVTs), and maternal immune cells (like regulatory T cells, NK cells, and macrophages) at the maternal–fetal interface. These interactions modulate immune responses to be less aggressive, promoting immune tolerance while still enabling protective immunity against infections, thus supporting the successful development of the fetus [22]. Extravillous trophoblasts (EVTs) contribute to this interaction by expressing immunomodulatory molecules, such as the human leukocyte antigen-G (HLA-G), which engage with maternal immune cells to inhibit their cytotoxic activity and promote fetal tolerance [28].

Population diversity also plays a critical role in understanding alloimmune recurrent pregnancy loss (RPL), as genetic and environmental factors can significantly influence immune responses at the maternal–fetal interface. Variations in human leukocyte antigen (HLA) genes, which are key mediators of maternal–fetal immune interactions, differ across populations and can impact the likelihood of immune tolerance or rejection. For instance, certain HLA-G mismatches between the mother and the fetus may increase the risk of RPL in some populations [29]. Additionally, the prevalence of immune conditions, such as elevated natural killer (NK) cell activity or imbalances in T-helper cell subsets (Th1/Th2/Th17), may vary among ethnic groups due to genetic predispositions or differences in immune system regulation [30]. Environmental factors, including diet, infections, and exposure to toxins, can further modulate immune responses, adding another layer of complexity [31]. Therefore, it is crucial to consider population diversity in the study of alloimmune RPL to ensure that findings are generalizable and applicable to a wide range of patients. Future research should prioritize including diverse study populations to better understand the interplay of genetic, environmental, and immunological factors in RPL and to develop tailored interventions that address the needs of different demographic groups.

### 2.1. Extravillous Trophoblasts (EVTs)

In human pregnancy, fetal trophoblast cells differentiate into villous (VT) and extravillous trophoblasts (EVTs), forming the placenta, after which EVTs invade the decidua and myometrium. Following implantation, EVTs invade maternal tissues, including the spiral arteries, ultimately replacing the vascular lumen [28]. Thus, the feto-maternal interface is formed, and EVTs and maternal immune cells come into contact with each other. EVTs play a vital role in the placenta. Beyond their primary functions of transporting oxygen and nutrients to the fetus and removing carbon dioxide and metabolic waste, they also modulate the maternal immune response to prevent it from attacking the fetus [32]. The physiological interactions between EVTs and immune cells help create a tolerogenic environment that allows the semi-allogeneic fetus to coexist with the maternal immune system without triggering rejection. The maternal immune system also dynamically modifies itself to induce tolerance against fetal tissues [33]. Any dysfunction in EVTs or disturbances in their immune microenvironment can potentially lead to RPL.

**Figure 1:**
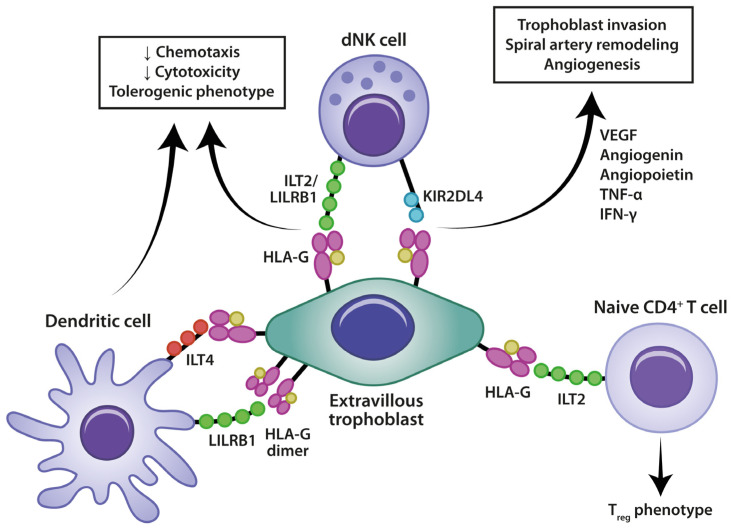
Tolerogenic immune interactions at the maternal–fetal interface: role of extravillous trophoblasts in regulating dNK cells. The figure illustrates the role of extravillous trophoblasts in interacting with maternal immune cells, promoting a tolerogenic environment critical for successful pregnancy. The extravillous trophoblast expresses HLA-G, a molecule that plays a pivotal role in modulating maternal immune responses. HLA-G interacts with receptors on decidual natural killer (dNK) cells, such as ILT2/LILRB1 and KIR2DL4, reducing dNK cell cytotoxicity and promoting a tolerogenic phenotype. These interactions support trophoblast invasion, spiral artery remodeling, and angiogenesis through the secretion of factors like VEGF, angiogenin, TNF-α, and IFN-γ via ILT4 and LILRB1 receptors, leading to reduced chemotaxis, decreased cytotoxicity, and the adoption of a tolerogenic phenotype. Naive CD4+ T cells are influenced by HLA-G through ILT2, driving their differentiation into regulatory T cells (Tregs), which are essential for maintaining immune tolerance and preventing the maternal immune rejection of the fetus. Together, these cellular interactions and immune modulations establish a supportive environment for implantation and pregnancy maintenance. dNK: decidual natural killer cell; HLA-G: human leukocyte antigen-G; ILT2/LILRB1: immunoglobulin-like transcript 2/leukocyte immunoglobulin-like receptor subfamily B1; ILT4: immunoglobulin-like transcript 4; KIR2DL4: killer immunoglobulin-like receptor 2DL4; Treg: regulatory T cells; VEGF: vascular endothelial growth factor; TNF-α: tumor necrosis factor-alpha; IFN-γ: interferon-gamma.

Unlike most somatic cells, which express classical major histocompatibility complex class I (MHC-I) molecules, such as human leukocyte antigen (HLA)-A or -B, EVTs express some unique immunomodulatory molecules, such as HLA-C, -E, -G, which interact with maternal immune cells to inhibit their cytotoxic activity [34,35]. HLA-G-expressing EVTs exert their inhibitory effects by binding to several receptors from immune cells. One such receptor is leukocyte Ig-like receptor subfamily B member 1 (LILBR1), or immunoglobulin-like transcript 2 (ILT2). ILT2 is an inhibitory receptor expressed on different immune cells, including NK cells, T cells, B cells, and antigen-presenting cells (APCs). It plays a critical role in maintaining immune tolerance and regulating immune responses. The interaction between EVTs and ILT2 from T cells inhibits their proliferation and chemotaxis, while also inducing the development of Tregs. Additionally, ILT2 stimulates the secretion of anti-inflammatory cytokines such as interleukin (IL)-10, IL-4, and transforming growth factor-beta (TGF-β), further promoting immune tolerance and regulating inflammatory responses [36]. In NK cells, ILT2 suppresses cytotoxicity and chemotaxis, while also inhibiting MICA/NKG2D expression, a potent activating immune receptor, as well as interferon-gamma (IFN-γ) secretion [37]. HLA-G-expressing EVTs can also interact with KIR2DL4 (CD158d), a unique member of the killer cell immunoglobulin-like receptor (KIR) family. KIR2DL4 is primarily expressed on NK cells and plays a dual role, functioning as both an activating and inhibitory receptor. Structurally, KIR2DL4 contains both an immunoreceptor tyrosine-based inhibitory motif (ITIM), which typically promotes inhibitory signaling, as well as motifs in the transmembrane domain that can associate with FcεRIγ, an adaptor protein that promotes activating signals. When NK cells interact with HLA-G via KIR2DL4, they are stimulated to secrete pro-inflammatory cytokines such as IFN-γ and tumor necrosis factor-alpha (TNF-α) [38]. Interestingly, this interaction does not trigger the usual NK cell cytotoxic response, thereby allowing them to promote tissue remodeling and immune modulation without harming the trophoblasts, which are crucial for the developing placenta. Polymorphisms in both KIR2DL4 and HLA-G have been linked to pregnancy complications, including preeclampsia and RPL [39,40] (Figure 1).

### 2.2. Natural Killer (NK) Cells

Natural killer (NK) cells originate from hematopoetic stem cells that initially differentiate down the lymphoid line, and represent a primary component of the innate immune system. Killer cell immunoglobulin-like receptors (KIRs) are critical components of the immune system, especially in regulating NK cell activity. These highly polymorphic receptors are mainly expressed on NK cells and help to distinguish between healthy and abnormal cells, such as those that are virus-infected or cancerous. KIRs interact with MHC class I molecules that are present on all nucleated cells, allowing NK cells to monitor cellular health and respond appropriately [41].

Uterine natural killer (uNK) cells are a unique subset of immune cells that reside in the uterus, and, depending on their location and the pregnancy status of the host, they can be categorized as either endometrial NK (eNK) cells or decidual NK (dNK) cells. uNK cells are a key component of the uterine mucosal immune system and play a crucial role in early pregnancy by regulating vascular function, trophoblast invasion, and placental development and immune tolerance. Unlike peripheral blood NK (pbNK) cells, which circulate throughout the body, uNK cells are specifically adapted to function within the uterine environment. Their primary role is to facilitate successful implantation and support fetal development while simultaneously protecting against pathogens [42,43]. uNK cells play an essential role in the early stages of vascular remodeling in the decidua and regulate the depth of invasion by EVTs, which are primarily responsible for arterial transformation during pregnancy. By the time the trophoblast completes its invasion around the 20th week of pregnancy, the number of uNK cells starts to decline, resulting in a much smaller uterine lymphocyte population compared to the first trimester [44].

uNK cells are identified by specific surface markers that distinguish them from other immune cells. The most notable marker is CD56, with uNK cells typically exhibiting a CD56^bright^ phenotype, which indicates that they are highly activated and functional. They also express CD49a, which is involved in adhesion to extracellular matrix, and CD69, an early activation marker indicating that they are ready to respond to signals from the surrounding tissue [42]. Another distinguishing feature of uNK cells is their relatively low cytotoxicity compared to pbNK cells. While pbNK cells are known for their ability to kill virus-infected or cancer cells, uNK cells primarily secrete cytokines and growth factors that promote tissue remodeling and angiogenesis. This functional adaptation is crucial for creating a nurturing environment for the developing embryo [43,45]. uNK cells contribute to placental development in the early stages of pregnancy development, as, after implantation, they secrete vascular endothelial growth factor (VEGF), angiogenin, and angiopoietin to ensure blood flow to the fetus [46]. Upon embryo implantation, uNK cells undergo further proliferation and differentiation, producing a diverse range of cytokines. They also express several inhibitory NK cell receptors (iNKRs), such as killer cell immunoglobulin-like receptors (KIRs) and leukocyte immunoglobulin-like receptors, as well as C-type lectin-like receptor families (NKG2/CD94) [47]. Changes in the cytokine secretion profile of dNK cells have been linked with various other pregnancy complications besides RPL, such as preeclampsia (PE) [48] and intrauterine fetal growth restriction (IUFGR) [43].

From a functional perspective, KIRs can be classified as either activating or inhibitory, depending on their effect on uNK cells. This classification is primarily determined by the structure of their cytoplasmic tails, where KIRs with short cytoplasmic tails act as activators, while those with long tails function as inhibitors [49]. Activating KIRs, due to their short cytoplasmic tail, lack immunoreceptor tyrosine-based inhibitory motifs (ITIMs) and instead transmit activating signals to uNK cells through association with adaptor proteins like TYROBP (a tyrosine kinase-binding protein, also known as DAP12), which contain immunoreceptor tyrosine-based activation motifs (ITAMs). These signals can promote the cytotoxic activity and cytokine production of uNK cells, thereby enhancing immune responses. Inhibitory KIRs possess a long cytoplasmic tail that contains ITIMs, which transmit inhibitory signals when the KIR binds to its ligand, typically a class I MHC molecule like HLA-C. These inhibitory signals dampen uNK cell activity, reducing their cytotoxic function and promoting immune tolerance, especially during pregnancy [50]. Based on the predominance of these activating or inhibitory KIRs, two primary KIR haplotypes have been defined: KIR A and KIR B. The KIR A haplotype is characterized by a predominance of inhibitory KIRs such as KIR2DL1, KIR2DL3, and KIR3DL1; it is associated with stronger inhibitory signals that promote immune tolerance, but may also be linked to complications in pregnancy, such as preeclampsia, due to insufficient uNK cell activation and poor trophoblast invasion. The KIR B haplotype is dominated by activating KIRs such as KIR2DS1 and KIR2DS2, which promote uNK cell activation; this haplotype is associated with more balanced or even stronger activating signals that enhance uNK cell function, improving trophoblast invasion and reducing the risk of pregnancy complications like preeclampsia. Therefore, this balance between activating and inhibitory KIRs plays a key role in determining the outcomes of immune responses during pregnancy [50,51].

**Figure 2:**
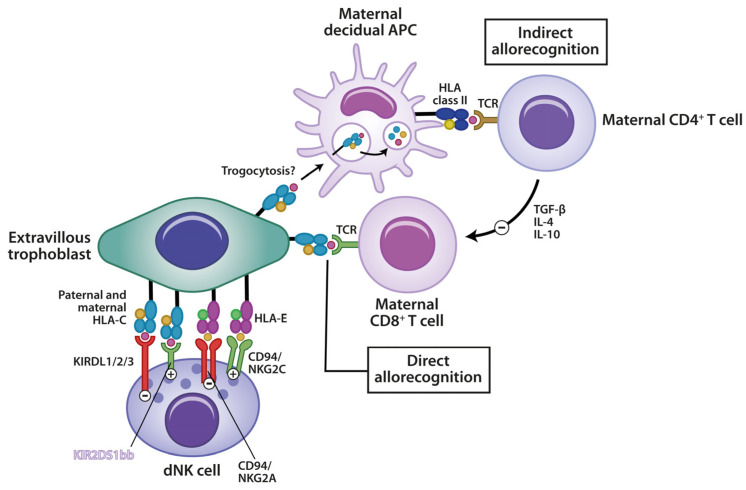
Immune recognition at the maternal–fetal interface: interactions between extravillous trophoblasts and maternal immune cells. This figure illustrates the immune interactions at the maternal–fetal interface during pregnancy, highlighting how maternal immune cells recognize fetal antigens. The extravillous trophoblasts (fetal cells) express both paternal and maternal HLA-C antigens that interact with dNK cells via KIR receptors, as well as non-classical MHC molecules like HLA-E, which engage with receptors on decidual natural killer (dNK) cells, including KIRDL1/2/3, CD94/NKG2A, and NKG2C receptors. These interactions modulate dNK cell activity, promoting a tolerogenic environment that supports implantation and placental development. The direct recognition of these HLA molecules by maternal CD8+ T cells, through T cell receptors (TCRs), represents a pathway of direct allorecognition, contributing to the immune system’s regulation at the maternal–fetal interface. Additionally, maternal antigen-presenting cells (APCs) process fetal antigens from EVTs, potentially through trogocytosis (the transfer of membrane components). These processed antigens are presented to maternal CD4+ T cells via HLA class II molecules, leading to indirect allorecognition. This interaction induces a regulatory immune response characterized by the production of tolerogenic cytokines, such as TGF-β, IL-4, and IL-10, which help suppress inflammation and protect the fetus from maternal immune rejection. In contrast, direct allorecognition occurs between maternal CD8+ T cells and fetal antigens. APC: antigen-presenting cell; TCR: T cell receptor; HLA-C: human leukocyte antigen-C (paternal and maternal); HLA-E: human leukocyte antigen-E; KIR: killer immunoglobulin-like receptors; dNK: decidual natural killer cells; CD8+ T cells: cytotoxic T cells involved in direct allorecognition; CD4+ T cells: helper T cells involved in indirect allorecognition; TGF-β: transforming growth factor-beta; IL-4, IL-10: interleukins.

As previously mentioned, uNK cells specifically bind to non-classical class I MHC molecules on trophoblast cells, playing a key role in mediating immune recognition and promoting immune tolerance toward the embryo. The killer immunoglobulin-like receptor (KIR) on the NK cell surface, which detects class I MHC, limits NK cell cytotoxicity and contributes to maintaining immune tolerance balance (Figure 2). In addition to the previously discussed interactions between uNK cells and HLA-G, NK cells can also recognize HLA-E on EVTs through their NKG2A/CD94 receptor complex, leading to inhibitory signals and thereby maintaining immune tolerance at the maternal–fetal interface; on the other hand, by binding to the NKG2C/CD94 complex, they can induce activatory signals [52,53].

Furthermore, uNK cells also specifically interact with HLA-C, a classical MHC class I molecule expressed on EVTs, which is also the only known polymorphic MHC molecule expressed on these cells. HLA-C1 and HLA-C2 epitopes are recognized by inhibitory receptors such as KIR2DL1 (for C2) and KIR2DL2/3 (for C1). When inhibitory KIRs on uNK cells bind to their respective HLA-C ligands, they transmit signals that suppress NK cell cytotoxicity, promoting an immune-tolerant environment necessary for fetal survival. Activating KIRs such as KIR2DS1 can also bind HLA-C2, leading to NK cell activation, which may contribute to pregnancy complications like RPL, preeclampsia, and IUFGR, underscoring the delicate balance required between NK cell activation and inhibition during pregnancy. As the embryo acquires HLA-C from both parents, the paternal genes would have a direct and major influence on uNK cell function and activation [54].

Two extreme interactions are known at present: one being the inhibitory KIR-A with HLA-C2, and the other being the activator KIR-B with HLA-C1. A maternal KIR AA genotype combined with a fetal HLA-C2 is associated with an increased risk of preeclampsia, as the HLA-C2 on the trophoblast interacts predominantly with the inhibitory KIR2DL1 receptor on uNK cells. This leads to the excessive inhibition of uNK cell activity, resulting in insufficient trophoblast invasion into the uterine spiral arteries. This insufficient invasion contributes to poor placental development and vascular remodeling, which are key features of preeclampsia. Interestingly, when the fetus is homozygous for HLA-C1 in combination with a maternal KIR AA genotype, there is no increased risk of preeclampsia. In this case, the interaction between HLA-C1 and the inhibitory KIR2DL2/3 receptor is not as strong, resulting in a less pronounced inhibitory signal to the uNK cells. This weaker inhibition allows for normal trophoblast invasion, preserving the development of the uterine arteries and preventing the onset of preeclampsia [50,55,56].

The complex interactions between maternal immune cells and the extravillous trophoblasts are shown in Figure 2.

### 2.3. Regulatory T Cells (Treg)

Regulatory T cells (Treg), characterized by a CD4^+^CD25^+^Foxp3^+^ phenotype, are a specialized subset of CD4+ T cells responsible for the self-tolerance of the immune system and prevention of autoimmune diseases [57]. Tregs suppress the activity of other immune cells such as effector T cells, dendritic cells, and macrophages in order to prevent excessive immune responses by various mechanisms such as the secretion of immunosuppressive cytokines like IL-10, TGF-β, and IL-35. Tregs can also act by directly inhibiting the proliferation of other T cells and limiting their ability to produce pro-inflammatory cytokines [58]. During pregnancy, T cells undergo physiological expansion and constitute 10–20% of decidual immune cells in the first trimester; however, not all of these are CD4^+^ Tregs [59,60,61]. As the embryo expresses paternally derived alloantigens, an inflammatory response can be triggered at the time of implantation, which might jeopardize the pregnancy, so the maternal immune system shifts toward an active state of tolerance, which is essential for a healthy pregnancy. This immune tolerance is largely mediated by Tregs, which help suppress the maternal immune response against the fetus, protecting it from immune rejection. Therefore, Tregs are crucial in creating an environment that fosters placental development and supports the sustainability of the pregnancy by controlling inflammation in the early phase of pregnancy and ensuring a receptive decidual environment [59]. If this mechanism does not occur, RPLs are observed [62].

The primary mechanism by which decidual Tregs exert their immunosuppressive effect involves the secretion of IL-10 and TGF-β, along with the expression of surface markers such as CD25, Cytotoxic T-Lymphocyte Antigen 4 (CTLA-4), and Programmed Death-Ligand 1 (PD-L1), mediators which inhibit effector T cells in the early stages of pregnancy. IL-10 is a potent anti-inflammatory cytokine that suppresses the activity of effector T cells and the production of pro-inflammatory cytokines, while also modulating the function of APCs such as dendritic cells, in order to reduce the likelihood of an aggressive immune response at the maternal–fetal interface. TGF-β is another key immunosuppressive cytokine that promotes immune tolerance by inhibiting T cell proliferation and differentiation, especially in Th1 and Th17 subsets; TGF-β also contributes to the generation of additional Tregs, thereby reinforcing the suppressive environment [58]. CD25, the high-affinity receptor for IL-2, is essential for the survival and expansion of Tregs. By capturing IL-2, Tregs limit its availability for effector T cells, thereby inhibiting their proliferation [63]. CTLA-4 is an inhibitory receptor that downregulates the activation of T cells by binding to CD80 and CD86 on APCs, preventing them from fully activating effector T cells [64]. PD-L1 is an immune checkpoint molecule that interacts with PD-1 on T cells to inhibit their activation and promote immune tolerance. Therefore, the PD-L1/PD-1 axis is key for maintaining a non-inflammatory environment at the maternal–fetal interface [65].

Both quantitative and qualitative issues with regulatory T cells (Tregs) can lead to significant complications in pregnancy and immune system dysfunction [59]. Low circulating levels of Tregs have been found to be predictive of miscarriage risk, particularly in newly pregnant women with a history of reproductive failure. These lower Treg levels are associated with an impaired ability to establish maternal–fetal tolerance, leading to an increased likelihood of immune-mediated pregnancy loss [66,67,68]. Interestingly, recent studies have introduced a novel perspective by suggesting that both lower and higher levels of Tregs can increase miscarriage risk, indicating a U-shaped distribution of Treg effects on pregnancy outcomes [68,69]. This pattern implies that either an insufficient or an excessive number of Tregs can disrupt the immune balance required for successful pregnancy, which contrasts with earlier research focused predominantly on low Treg levels as the primary risk factor. While low Treg levels may fail to suppress maternal immune responses adequately, elevated Treg levels might paradoxically suppress the immune system too strongly, potentially hindering essential immune responses needed for healthy fetal development [61,70]. These findings contrast with earlier research, which predominantly focused on low Treg levels as the primary risk factor for RPL. By recognizing that both ends of the Treg spectrum may contribute to pregnancy complications, this expanded understanding of Treg dynamics highlights the importance of maintaining optimal immune homeostasis, where the balance—not simply the quantity—of Tregs is crucial for fostering maternal–fetal tolerance.

### 2.4. Other T Cell Subpopulations

**Figure 3:**
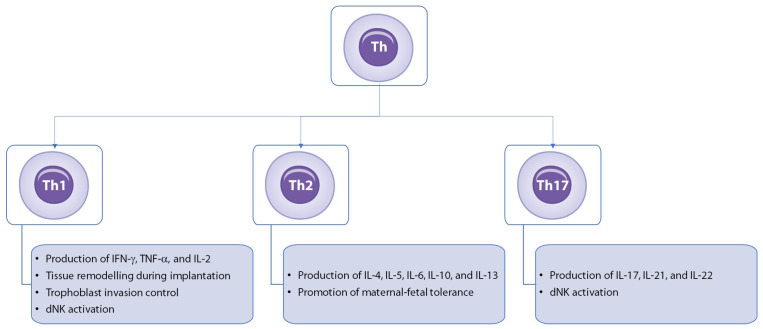
Roles of T-helper cell subsets in immune regulation and recurrent pregnancy loss. This figure illustrates the roles of T-helper (Th) cell subsets—Th1, Th2, and Th17—in pregnancy, emphasizing their distinct cytokine production and contributions to maternal–fetal interactions. Th1 cells produce IFN-γ, TNF-α, and IL-2, supporting tissue remodeling during implantation, regulating trophoblast invasion, and activating decidual natural killer (dNK) cells. Th2 cells secrete IL-4, IL-5, IL-6, IL-10, and IL-13, promoting maternal–fetal tolerance and creating an anti-inflammatory environment crucial for sustaining pregnancy. Th17 cells produce IL-17, IL-21, and IL-22, contributing to dNK cell activation but potentially posing risks when dysregulated. The balance between these Th cell subsets is essential for successful implantation, placental development, and pregnancy maintenance. Th: T-helper cell; IFN-γ: interferon-gamma; TNF-α: tumor necrosis factor-alpha; IL: interleukin; dNK: decidual natural killer cells.

Other T cell subpopulations, such as Th1, Th2, and Th17 cells, have also been the focus of extensive research in the context of RPL. Helper T cells are a group of T lymphocytes that generally play a supportive role in the immune response. They can be best described based on the cytokines they secrete and, consequently, the type of immune responses they promote (Figure 3).

Th1 cells secrete IFN-γ, IL-2, and TNF-α, which are crucial for activating macrophages and promoting cell-mediated immunity, especially against intracellular pathogens. In the case of pregnancy-related complications, Th1 cells are recruited to the endometrium, decidua, and placenta where they play a significant role in inflammation by producing pro-inflammatory cytokines that can activate NK cells in the decidua. The excessive recruitment and activation of the Th1-NK cell axis disturb the delicate immune balance needed to support pregnancy, favoring pro-inflammatory responses over immune tolerance [71]. In contrast, Th2 cells produce IL-4, IL-5, IL-6, IL-10, and IL-13, which promote humoral immunity, activating B cells and supporting antibody production.

Th17 cells are a specialized subset of CD4+ T cells that differentiate in the presence of IL-1β, IL-6, IL-23, and TGF-β. Interestingly, TGF-β has a dual role in the immune system. In the presence of IL-6 and IL-1β, TGF-β promotes the differentiation of Th17 cells. However, in the absence of these inflammatory cytokines, TGF-β instead promotes Treg differentiation [72]. Therefore, TGF-β acts as a context-dependent cytokine, facilitating Th17 differentiation when pro-inflammatory signals are present, but steering cells towards immune tolerance via Tregs in their absence. Th17 cells are characterized by the secretion of IL-17, IL-21, and IL-22, which are involved in recruiting neutrophils and are, therefore, important for immune defense against extracellular bacteria and fungi, as well as contributing to inflammatory autoimmune diseases. Furthermore, Th1/Th2/Th17 and Treg cell lines have the ability to transform into one another [73]. Th17 cells play a critical role in the immune environment during pregnancy complications by inducing the activation of dNK cells. As previously described, once activated, dNK cells can impair the vascular reactivity of uterine arteries, leading to poor blood flow to the developing fetus. This disruption in vascular function compromises trophoblast invasion and placental development, ultimately contributing to RPL [74,75].

Th1 responses suppress Th2 responses and vice versa; when not in balance, they influence implantation and fetal development and differentiation. Studies have investigated the balance between Th1 and Th2 cells during pregnancy by measuring circulating levels of Th1 (IFN-γ, IL-2, TNF-α, IL-12) and Th2 (IL-4, IL-5, IL-6, IL-10, and IL-13) cytokines, or by examining the expression of Th1 (CXCR3) and Th2 (CCR4) chemokine receptors. Pregnancy has long been viewed as a Th2-dominant state [76,77], supported by evidence showing a shift towards anti-inflammatory cytokines and improvement in Th1/Th17-mediated autoimmune disorders like rheumatoid arthritis [78], while Th2-mediated conditions such as later-stage systemic lupus erythematosus can worsen [79]. Also, RPL has often been associated with various autoimmune diseases where disruptions in immune regulation play a key role. Th1-related conditions such as Hashimoto’s disease [80], early-stage systemic lupus erythematosus [81], systemic sclerosis [82], and Sjogren’s syndrome [83,84] have all been linked to RPL. In these autoimmune diseases, a Th1-shifted immune response or an upregulation of Th17 cells has been reported, contributing to increased inflammation and immune dysregulation, which can negatively impact pregnancy and lead to miscarriage.

On the other hand, Th1 to Th2 shift is thought to be critical in protecting the fetus from rejection, as Th1 responses are typically pro-inflammatory and associated with cytotoxic immune responses, whereas Th2 responses promote humoral immunity and tolerance. The rise in anti-inflammatory cytokines like IL-10 and TGF-β early in pregnancy reflects this progressive shift, supporting the notion of pregnancy as an anti-inflammatory state. However, some studies have challenged this view, suggesting that both Th1 and Th2 cells are necessary to maintain a proper immune balance during pregnancy. Indeed, obstetrical complications such as RPL and preeclampsia have been associated with a predominant Th1 immune response [77,85], but, in some cases, RPL has also been linked to a shift towards Th2 immunity [86]. This suggests that both immune imbalances—either Th1 dominance or inappropriate Th2 shifts—can contribute to pregnancy complications.

### 2.5. B Cells

B cells are important components of the adaptive immune system, responsible for antibody production and the regulation of immune responses. They undergo significant adaptations during pregnancy, which are characterized by lymphopenia, particularly in its second half [87]. Changes in B cell subpopulations and their functions have been associated with adverse pregnancy outcomes, including RPL. Several studies observed a significant increase in the percentage of CD19^+^ B cells in women experiencing first-trimester RPL [87,88]. However, contrasting findings have also been reported: decreased percentages of CD19^+^ B cells in women who have experienced RPL compared to healthy pregnancies [87,89]. This discrepancy suggests that B cell regulation and function may vary depending on pregnancy status and could indicate different underlying mechanisms of immune dysfunction in RPL. A recent systematic review highlighted the association between modified B cell proportions and RPL, suggesting that the dysregulation of these cells may hinder the successful implantation and maintenance of a pregnancy [87]. Altogether, these variations in the results of studies observing peripheral B cell populations highlight the need for further research to fully understand the role of B cells in pregnancy loss [90].

The immunological landscape during pregnancy is complex, with B cells exhibiting both pro-inflammatory and regulatory functions. For instance, studies have shown that uterine B cells can gain regulatory characteristics that are essential for successful implantation and fetal development [91]. B cells, especially regulatory B cells (Bregs), play a crucial role in promoting maternal immune tolerance during pregnancy, contributing to tolerance primarily through the production of the anti-inflammatory cytokine IL-10. Additionally, Bregs can induce tolerance through the CD39/CD73 pathway, which involves the production of adenosine, a molecule with potent immunosuppressive properties which suppresses the activation of effector T cells and other immune cells. CD39 and CD73 are enzymes expressed on the surface of Bregs that convert adenosine triphosphate (ATP), a pro-inflammatory molecule, into adenosine [92]. This is further supported by findings that indicate lower levels of IL-10 and other anti-inflammatory molecules in women experiencing pregnancy loss compared to those with successful pregnancies [93].

The presence of autoantibodies, such as anticardiolipin antibodies produced by B cells, has also been implicated in RPL. Research has established a correlation between these antibodies and defective placental development [94]. In a cohort study, a significant proportion of women who experienced RPL tested positive for these antibodies, underscoring the potential role of B cell-mediated autoimmune responses in pregnancy loss [95].

The interplay between B cells and Tregs is also vital for maintaining immune tolerance during pregnancy. As Tregs are known to suppress excessive immune responses, their dysfunctions, which are often associated with B cell abnormalities, can lead to RPL [96].

## 3. Limitations and Constraints

While significant progress has been made in understanding immune mechanisms involved in RPL, several methodological limitations continue to constrain research in this field. One key challenge is the inherent variability in immune responses among individuals. Immune cell composition and function can vary greatly depending on genetic, environmental, and lifestyle factors, making it difficult to generalize findings across diverse populations [97]. This variability introduces challenges in interpreting study results, as immune responses may not be uniform across individuals or even within the same individual at different stages of pregnancy [98].

Another major constraint is the limited accessibility of human decidual tissues for research purposes. The decidua plays a critical role in establishing maternal–fetal tolerance; yet, ethical and practical limitations restrict the ability to obtain and study these tissues extensively. Most studies must rely on samples from elective terminations or miscarriages [99,100], which may not fully represent immune conditions during healthy pregnancies. Additionally, animal models, while valuable, cannot completely replicate the unique immunological environment of human pregnancy, further complicating the extrapolation of findings [101,102]. Addressing these limitations will require developing new less invasive sampling methods and refining models that more accurately mimic human reproductive immunology.

## 4. Therapeutic Approaches

The clinical management of RPL remains both challenging and limited due to insufficient evidence. For couples trying to conceive and suffering from RPL, the therapeutic approaches are aimed at inducing a modulatory effect on the immune system to increase the chance of a healthy pregnancy. While therapies such as intravenous immunoglobulin (IVIg), liposomes, lymphocyte immunization therapy (LIT), anticoagulants (including low-dose aspirin or low-molecular-weight heparin), glucocorticoids, immunosuppressants, TNF-α inhibitors, and intralipid infusions have been explored (Table 1), their results have not been completely satisfactory and many challenges remain in achieving consistent long-term success in preventing RPL. Therefore, their routine use is not currently recommended [2]. Instead, it is advised that patients with unexplained RPL consider participation in clinical research programs tailored to their individual circumstances.

Treatments such as intravenous immunoglobulin (IVIg) aim to modulate immune activity primarily by suppressing NK cell overactivity and enhancing Treg function. However, the mechanisms underlying the therapeutic effects of IVIg are complex, and it is unlikely that a single mechanism fully explains its benefits. Despite its widespread use as an immuno-modulating therapy for over 30 years, there remains limited understanding of the factors that determine its effectiveness. At present, there is no standardized policy or protocol defining the optimal dosage or frequency for administering IVIg. While some studies [103,104] found no significant effect of IVIg on pregnancy outcomes in women with RPL, several more recent meta-analyses and studies support its use, particularly in those patients with RPL that exhibit immune abnormalities such as elevated NK cell levels and cytotoxicity, imbalanced Th1/Th2 ratios, or the presence of autoantibodies [105,106,107,108,109,110]. Evidence also suggests that pre-conception or early-in-conception IVIg treatment is more effective than post-conception administration, as establishing and maintaining an anti-inflammatory environment prior to conception produces better outcomes for implantation and the continuation of a pregnancy [108,111]. These newer findings have led to a significant change in the ESHRE guidelines regarding IVIg use in RPL: while the ESHRE 2017 guideline did not recommend IVIg as a treatment in RPL [3], the ESHRE 2022 guideline states that “the use of repeated and high doses of IvIg very early in pregnancy may improve live birth rate in women with four or more unexplained RPL” [2]. However, while these recent studies indicate the potential benefits of IVIg, the significant heterogeneity in study populations limits the strength of recommendations for its routine use.

Glucocorticoids are generally used to suppress inflammation by inhibiting pro-inflammatory cytokine production and reducing T cell activation, making them particularly useful in autoimmune-related RPL, such as those exhibiting antinuclear antibodies, anti-phospholipid antibodies, or antithyroid antibodies. Challenges include selecting the appropriate glucocorticoid, determining optimal dosages, and defining treatment duration. Current recommendations suggest using short-acting glucocorticoids like prednisolone at low doses (≤10 mg/day) to minimize risks, while avoiding long-acting options such as dexamethasone and betamethasone due to their potential adverse effects on fetal development. Limited evidence indicates that a pre-conception course of 1–3 months may yield better outcomes than post-conception initiation, but further research is needed to substantiate these findings [112]. The use of prednisolone as a treatment for RPL, particularly in women with elevated uNK cell counts, has been a topic of significant interest and debate. Evidence suggests that pre-conception treatment with prednisolone can reduce uNK cell numbers, with studies reporting a potential improvement in live birth rates [112,113]. Additionally, randomized controlled trials (RCTs) have demonstrated increased rates of ongoing pregnancies at 20 weeks’ gestation with prednisolone treatment when combined with aspirin and heparin [114]. However, these studies often lack data on live birth rates, adverse effects, and the independent impact of prednisolone due to the presence of co-treatments. Despite the potential benefits of low-dose prednisolone, uncertainties remain due to variations in the definition of RPL, inconsistent control groups, and the unclear relationship between uNK cells and peripheral NK cells. Confounding factors, such as co-medications, further complicate interpretation. The ESHRE has emphasized the need for high-quality adequately powered RCTs to evaluate the efficacy and safety of prednisolone in RPL management [2]. While prednisolone appears to be a promising option, its routine use cannot currently be recommended without more robust evidence. Future research must address these limitations to establish clearer guidelines for its application in clinical practice.

Low-dose aspirin and heparin are frequently prescribed to address thrombosis-related pregnancy loss, especially in cases involving antiphospholipid syndrome (APS), as they improve placental blood flow and reduce the risk of clot formation [115,116,117,118]. Moreover, this dual therapy may benefit women with unexplained RPL by addressing potential subclinical thrombophilic conditions that may not meet diagnostic criteria for APS but still impair placental function. Despite its widespread use, the optimal timing, dosage, and duration of aspirin and heparin therapy remain areas of ongoing research, particularly in cases of RPL without clear evidence of thrombophilia, where the efficacy of such treatment is less established. However, the ESHRE 2022 guideline for RPL does not recommend low-dose aspirin and/or heparin in women with unexplained RPL, as they do not seem to improve live birth rate [2].

Other immunomodulatory therapies, such as TNF-α inhibitors, have shown promise in treating RPL associated with autoimmune conditions. Elevated levels of TNF-α have been strongly associated with an increased risk of miscarriage, highlighting its significance in RPL [119,120,121]. TNF-α inhibitors, which neutralize the activity of TNF-α, have traditionally been reserved for pregnant women with autoimmune conditions such as rheumatoid arthritis (RA) or inflammatory bowel disease (IBD) [122]. However, recent evidence suggests that these immunosuppressive agents may also benefit women with refractory RPL by mitigating inflammation and improving pregnancy outcomes. Clinical trials exploring TNF-α inhibitors for RPL have provided mixed but encouraging results. Etanercept has been the most frequently studied TNF-α inhibitor in this context, demonstrating higher rates of healthy deliveries compared to adalimumab in some studies due to its large molecular size, reducing the risk of fetal exposure until later gestation [123]. Adalimumab and certolizumab pegol have also been successfully used in clinical trials for RPL, showing promising results without significant adverse maternal, fetal, or neonatal outcomes [66]. The safety of TNF-α inhibitors during pregnancy has been extensively studied in other contexts, such as RA and IBD, where these drugs have demonstrated no significant increase in spontaneous abortion or adverse pregnancy outcomes. Observational studies and clinical trials in women with autoimmune diseases receiving TNF inhibitors have reported successful ovulation induction, conception, and healthy deliveries [124]. However, recent studies suggest that TNF inhibitors might carry a moderate risk of adverse pregnancy outcomes, warranting careful consideration and monitoring in clinical use [119,125]. Despite these promising outcomes, most studies evaluating TNF-α inhibitors in RPL are limited in sample size, lack long-term follow-up, and vary in methodology, making it challenging to draw definitive conclusions. In summary, while TNF-α inhibitors hold promise as a treatment option for RPL, particularly in cases associated with immune dysfunction, further high-quality studies with larger cohorts and standardized methodologies are necessary. Long-term follow-up and a deeper understanding of potential complications will be critical to refine their role in managing RPL and ensuring their safety and efficacy in this population. The ESHRE 2022 guideline for RPL makes no reference to TNF-α inhibitors as a possible treatment option [2].

Lymphocyte immunization therapy (LIT) has emerged as a potential treatment for RPL by modulating maternal immune responses to create a more tolerogenic environment [17]. This process involves injecting peripheral white blood cells, typically derived from the male partner, into the prospective mother to enhance her immune system’s ability to tolerate the paternal antigens of the fetus [126]. While the exact mechanisms underlying LIT’s effects are not fully understood, several hypotheses suggest that it promotes the production of anti-paternal cytotoxic antibodies (APCAs), anti-idiotypic antibodies (Ab2), and mixed lymphocyte reaction-blocking antibodies (MLR-Bf). These antibodies are thought to shield the fetus from the maternal immune system, downregulate maternal IL-2 receptors, and inhibit T cell activation, thereby reducing immune-mediated pregnancy loss. LIT also appears to shift the maternal immune response towards a Th2-dominant profile, decrease NK cell activity, and improve the Treg population, thereby fostering an immune environment conducive to implantation and pregnancy maintenance [17,127,128]. Additionally, the use of paternal lymphocytes has demonstrated superior outcomes over autologous or third-party lymphocytes, as paternal cells more effectively stimulate the production of protective antibodies and immune factors [127]. Several studies have examined the safety and efficacy of LIT [20,129]. Recent meta-analyses indicate that LIT not only improves live birth rates, but also demonstrates a favorable safety profile [130,131]. Adverse reactions to LIT are generally mild, with patients occasionally reporting localized pain and swelling at the injection site [132]. Despite its promising results, the optimal administration route and dosing regimen remain under investigation, emphasizing the need for further research to standardize protocols and optimize outcomes. Current evidence supports LIT as both an effective and safe option for managing RPL, offering hope to couples struggling with repeated miscarriages. The ESHRE 2022 guideline for RPL states that LIT “should not be used as treatment for unexplained RPL as it has no significant effect and there may be serious adverse effects” [2].

Cyclosporin A (CsA) is a macrolide immunosuppressant widely used to prevent allograft rejection and manage autoimmune diseases due to its ability to regulate T cell, NK cell, and monocyte functions [133]. Recent studies suggest that low-dose CsA may have additional benefits in the context of pregnancy, particularly in supporting trophoblast function and enhancing maternal–fetal immune tolerance [134,135,136]. In terms of safety, the U.S. Food and Drug Administration (FDA) classifies CsA as a category C drug [137], with evidence from animal studies suggesting that only higher doses (2–10 mg/kg/day) might adversely affect the fetus [138]. Clinical studies on low-dose CsA in women with unexplained recurrent pregnancy loss (RPL) undergoing in vitro fertilization (IVF) and embryo transfer have reported increased live birth rates without maternal or fetal adverse events. However, a meta-analysis indicated a potential association between CsA use and an increased risk of preterm birth, emphasizing the need for further research to establish the long-term safety of CsA for both mothers and their offspring [134]. Additionally, the optimal timing for CsA withdrawal during pregnancy remains unclear, with studies suggesting varying durations ranging from 8 to 28 weeks of gestation [113]. More clinical trials are needed to define the therapeutic duration and evaluate the long-term consequences of CsA use during pregnancy. Tacrolimus, another immunosuppressive drug and calcineurin inhibitor, has gained attention for its safety and efficacy in pregnancy. Originally used to reduce the risk of organ rejection in transplant recipients, tacrolimus has been shown to improve live birth rates in women with RPL and elevated Th1/Th2 cell ratios [139]. Also classified as a category C drug by the FDA, tacrolimus has demonstrated a reassuring safety profile in pregnant women, with numerous reports of successful post-transplant pregnancies [140,141,142]. These findings suggest that tacrolimus may offer a viable option for managing immune-related RPL. Sirolimus, also known as rapamycin, is an immunomodulatory agent approved for use in solid organ transplantation and known for its anti-tumor properties [55]. Sirolimus exerts its immunosuppressive effects through the inhibition of the mammalian target of the rapamycin (mTOR) kinase pathway, which disrupts co-stimulatory signals required for T and B cell proliferation [143]. Additionally, sirolimus promotes regulatory T cell (Treg) expansion, inhibits Th17 differentiation, and reduces inflammatory responses [144]. Sirolimus has been shown to suppress immune responses and improve reproductive outcomes in women with recurrent implantation failure by modulating the Th17/Treg axis [145]. In conclusion, immunosuppressive agents such as CsA, tacrolimus, and sirolimus hold promise for improving pregnancy outcomes in women with immune-related RPL by modulating the immune system to foster a tolerogenic maternal–fetal environment. While these agents have shown efficacy and safety in specific contexts, additional high-quality clinical trials are needed to standardize their use, define optimal dosing regimens, and confirm their long-term safety profiles for both mothers and their offspring. Immunosuppressive drugs are not mentioned in the ESHRE 2022 guideline for RPL [2].

Intralipid infusion is another therapy that has been explored for managing recurrent pregnancy loss (RPL), particularly in cases where immune dysfunction, such as elevated natural killer (NK) cell activity or Th1/Th2 cytokine imbalances, is suspected [146]. It involves intravenously administering a fat emulsion containing soybean oil, glycerin, and egg phospholipids [147]. Originally developed as a parenteral nutrition solution, its immunomodulatory properties have made it a candidate for treating certain pregnancy complications. Intralipid is thought to reduce NK cell cytotoxicity, modulate cytokine production, and impair macrophage antigen presentation [146,148]. Research on intralipid infusion for RPL has yielded mixed results. Some studies report improved pregnancy outcomes in select populations, particularly those with immunological risk factors like elevated NK cell activity [149,150]. Other studies, however, have found no significant improvement in live birth rates, suggesting limited efficacy in broader RPL populations [147]. On the other hand, intralipid is considered relatively safe, with minimal reported maternal or fetal adverse effects. Common side effects are mild and include localized pain or swelling at the infusion site [150]. Its safety profile and lower cost compared to IVIg make it an attractive option for some patients [146,150]. Despite its theoretical benefits and safety, intralipid is not routinely recommended for RPL due to inconsistent evidence of efficacy. It is often reserved for cases where other treatments have failed, and immunological abnormalities are suspected. Further high-quality large-scale studies are needed to determine its precise role and effectiveness in managing RPL. The ESHRE 2022 guideline for RPL states that “there is insufficient evidence to recommend intralipid therapy for improving live birth rate in women with unexplained RPL” [2].

Altogether, these therapies target the immune system at multiple levels, aiming to correct the underlying immune dysregulation that contributes to RPL. However, alloimmune RPL remains a challenging area of research, often categorized under unexplained RPL.

Personalized medicine is gaining recognition as a promising strategy for managing recurrent pregnancy loss (RPL), emphasizing the customization of treatments based on individual immunological profiles. Cutting-edge technologies, such as high-throughput sequencing and multi-omics analysis, provide valuable insights into the genetic, epigenetic, and environmental factors that shape immune responses, creating opportunities for the development of targeted and precise immunotherapies [99,151].

Recent advancements include novel therapies such as multivitamin supplementation, notably vitamin D, which has shown some effectiveness in RPL patients, though dosing inconsistencies remain [152,153]. Mesenchymal stem cell (MSC)-based therapies have also shown promise in animal models, improving pregnancy outcomes by modulating immune responses at the maternal–fetal interface and increasing uterine dendritic cell (uDC) frequency [154,155]. Despite these developments, the lack of a comprehensive understanding of RPL mechanisms continues to limit effective treatment options, underscoring the need for further research in this field.

Future studies should prioritize long-term follow-up to evaluate the safety and efficacy of immunomodulatory treatments, particularly regarding risks like infections or immune overactivation. Collaboration between researchers, clinicians, and patients is critical to translating research into effective clinical strategies, offering hope for reducing RPL and improving pregnancy outcomes [154,155].

**Table 1:** Summary of the various therapeutic approaches for RPL attributed to immunological causes, particularly those involving natural killer (NK) cells, T cells, and B cells.

Therapeutic Approach	Mechanism of Action	Target Population	Effectiveness
Intravenous immunoglobulin (IVIg) [2,103,104,105,106,107,108,109,110,111,156,157]	Modulates immune response, decreases B cell responses and NK cell activity and toxicity, and induces regulatory T cells, enhancing fetal tolerance	Women with RPL, especially with elevated NK cell activity	Moderate efficacy in reducing RPL; some variability in responses
Glucocorticoids (prednisone, prednisolone) [111,112,158]	Reduces inflammation by suppressing T cell activation and inhibiting pro-inflammatory cytokine production, thereby improving trophoblast proliferation and invasion	Women with autoimmune-related RPL, such as lupus or APS	Effective in reducing inflammation and improving outcomes in autoimmune-related RPL; long-term use requires monitoring; currently not usually recommended due to significant adverse effects
Low-dose aspirin [115,116]	Reduces thrombotic events, which helps in improving placental blood flow	Women with RPL and thrombotic complications or placental insufficiency	Effective in reducing RPL risk related to placental insufficiency; commonly used with heparin
Heparin [115,116,117,118]	Prevents clot formation by inhibiting thrombin, protecting against APS-related RPL	Women with APS or clotting disorders leading to RPL	Highly effective in women with APS; standard of care for RPL related to clotting disorders; no significant effects on unexplained RPL
TNF-α inhibitors (e.g., infliximab, etanercept, adalimumab) [66,120,121,123]	Blocks TNF-α, a pro-inflammatory cytokine involved in immune activation and inflammation	Women with high levels of TNF-α or autoimmune diseases contributing to RPL	Promising results in reducing miscarriage risk in autoimmune-related RPL, though more research is needed
Lymphocyte immunization therapy (paternal leukocyte immunization) [127,128,130,131,159]	Induces maternal tolerance to fetal antigens by exposing the mother to paternal leukocytes	Women with unexplained RPL, often used in immunotherapy trials	Effectiveness is variable; may be beneficial in certain cases of unexplained RPL, but not widely used
Immunosuppressants (cyclosporine, tacrolimus, sirolimus) [135,136,141,142,145]	Inhibits T cell activation by suppressing IL-2 production, reducing immune-mediated fetal rejection	Women with RPL linked to heightened T cell activity or autoimmune conditions	Shows potential effectiveness in T cell-mediated immune responses, but data are limited and more studies are needed
Intralipid infusion [146,147,148,149,150]	Provides fatty acids that may modulate immune function and reduce NK cell activity	Women with RPL and high NK cell activity	Moderate effectiveness; some evidence supports efficacy, but results are mixed; often used in conjunction with other therapies

## 5. Conclusions

The immunological causes of RPL highlight the complexities of the maternal immune system’s role in pregnancy, where a delicate balance between immune tolerance and protection is crucial for maintaining fetal development. The involvement of immune cells such as NK, T, and B cells has been well documented. When their balance is disturbed, as seen in the overactivation of NK cells, the improper functioning of Th1 and Th17 cells, or the production of autoantibodies by B cells, the immune system may initiate harmful inflammatory responses or fail to develop the necessary tolerance to the fetus.

Research into the immune mechanisms of RPL has provided valuable insights into the distinct roles played by these immune cells. NK cells, particularly in the decidua, are essential for trophoblast invasion and placental vascular remodeling. Overactive NK cell responses, driven by imbalances in cytokine production, can disrupt these processes, leading to pregnancy failure. Similarly, the pro-inflammatory responses generated by Th1 and Th17 cells can further exacerbate the situation by inducing inflammation that harms the fetal environment, while a reduction in Treg cells, which are crucial for maintaining immune tolerance, further increases the risk of miscarriage.

B cells, and specifically Bregs, play a more subtle but equally important role in modulating immune responses during pregnancy. The increased presence of CD19^+^ B cells in some cases of RPL also suggests a more active role of B cells in immunological complications.

The various therapeutic strategies designed to address these immune dysfunctions reflect the importance of targeting the immune system to restore balance.

In conclusion, the immunological underpinnings of RPL are complex and multifactorial, involving the interplay of various immune cells and cytokine networks. Effective treatment requires a tailored approach that addresses the specific immune dysfunctions present in each case. As research advances, a more comprehensive understanding of the immune mechanisms involved in RPL will likely lead to improved therapeutic options, offering hope for better pregnancy outcomes in women affected by immunologically mediated RPL. To further advance the field, future research should prioritize identifying biomarkers that can reliably predict immune-related RPL risk early in pregnancy, allowing for timely intervention. Studies should also focus on refining immunological treatments to optimize safety and efficacy, including exploring personalized approaches that tailor therapies to individual immune profiles. Clinical practice would benefit from integrating routine immunological assessments in cases of RPL, aiding in the early detection of immune imbalances that could contribute to miscarriage. Establishing standardized guidelines for the use of therapies like IVIg, TNF-α inhibitors, or lymphocyte immunization therapy is essential to ensure consistency in patient care. By combining these research and clinical efforts, we can develop more precise evidence-based approaches to managing immunologically mediated RPL, ultimately improving outcomes for affected patients.

## Data Availability

Not applicable.
